# Growth differentiation factor 11 inhibits adipogenic differentiation by activating TGF‐beta/Smad signalling pathway

**DOI:** 10.1111/cpr.12631

**Published:** 2019-04-30

**Authors:** Hongke Luo, Yuchen Guo, Yuting Liu, Yuan Wang, Rixin Zheng, Yu Ban, Lin Peng, Quan Yuan, Weiqing Liu

**Affiliations:** ^1^ State Key Laboratory of Oral Diseases, National Clinical Research Center for Oral Diseases, West China Hospital of Stomatology Sichuan University Chengdu China

**Keywords:** 3T3‐L1 pre‐adipocytes, adipogenesis, growth differentiation factor 11, mesenchymal stem cells, TGF‐beta

## Abstract

**Objectives:**

Growth differentiation factor 11 (GDF11), an emerging secreted member of the TGF‐beta superfamily, plays essential roles in development, physiology and multiple diseases; however, its role during adipogenic differentiation and the underlying mechanisms remains poorly understood.

**Materials and methods:**

Bone marrow‐derived human mesenchymal stem cells (hMSCs) and 3T3‐L1 pre‐adipocytes were induced with adipogenic culture medium supplementing with different concentrations of recombinant GDF11 (rGDF11 0, 10, 50, 100 ng mL^−1^). Oil Red O staining, qRT‐PCR analysis, Western blot analysis and immunofluorescence staining were performed to assay adipogenesis.

**Results:**

For both hMSCs and 3T3‐L1 pre‐adipocytes, the presence of rGDF11 leads to a dose‐dependent reduction of intracellular lipid droplet accumulation and suppressed adipogenic‐related gene expression. Mechanically, GDF11 inhibits adipogenesis by activating Smad2/3‐dependent TGF‐beta signalling pathway, and these inhibitory effects could be restored by SB‐431542, a pharmacological TGF‐beta type I receptor inhibitor.

**Conclusions:**

Taken together, our data indicates that GDF11 inhibits adipogenic differentiation in both hMSCs and 3T3‐L1 pre‐adipocytes by activating Smad2/3‐dependent TGF‐beta signalling pathway.

## 1 INTRODUCTION

Growth differentiation factor 11 (GDF11), a novel TGF‐beta superfamily member, was shown to be crucial during development, physiology and multiple diseases.^1^ There has been accumulating evidence that GDF11 might be involved in adipogenesis. The expression of GDF11 was shown to decrease in the skeletal muscle of obese mice.^2^ Meanwhile, in women older than 60, GDF11 was reported to be negatively correlated with body mass, body mass index and fat mass.^3^ In addition, GDF11 levels were found to be higher in 12‐to 16‐year‐old girls with anorexia nervosa, which is characterized by low body mass.^4^ However, its effects on mesenchymal stem cell and pre‐adipocyte adipogenic differentiation and its underlying mechanism remain ambiguous.

Adipogenesis involves adipogenic lineage determination and terminal differentiation of the progenitor cells into mature adipocytes. Regarding the role of GDF11 in lineage commitment, Calif's laboratory demonstrated that GDF11 could determine whether stem cell adopts a glial or neuronal fate.^5^ Mallo's group just reported that the progenitor cell fate of *Gdf11* mutant embryos was shifted towards neural lineage.^6^ Furthermore, GDF11 inhibition was implicated to be a novel therapeutic strategy to promote erythroblastic differentiation.^7^ In terms of terminal differentiation, GDF11 was shown to exert an inhibitory effect on late‐stage erythropoiesis, and the blockade of GDF11 rescued terminal erythroid differentiation in turn.^8^

Previous studies of our laboratory uncovered the function of GDF11 in bone remodelling, demonstrating that GDF11 decreases bone mass by inhibiting the osteogenic potential of both MSCs and calvarial osteoblasts, while stimulating RANKL‐induced osteoclastogenesis of the haematopoietic precursors.^9^ Based on these intriguing observations, we further investigated its effect on chondrogenesis and found that GDF11 delays fracture healing by suppressing chondrocyte differentiation and maturation.^10^ Yet, whether GDF11 plays a part in adipogenic lineage commitment and pre‐adipocyte maturation remains elusive. Therefore, the aim of the present study was to unveil the possible impact of GDF11 on mesenchymal stem cell adipogenic differentiation and pre‐adipocyte maturation by culturing hMSCs and 3T3‐L1 cell line, respectively.

## 2 MATERIALS AND METHODS

### 2.1 Cell culture and differentiation

Human MSCs were purchased from American Type Culture Collection (ATCC) and cultured in alpha‐MEM (Gibco) supplemented with 10% foetal bovine serum (FBS; Gibco), plus 100 units mL^−1^ penicillin and 100 µg mL^−1^ streptomycin (Gibco), at 37°C with an atmosphere of 5% CO_2_. To induce adipogenic differentiation, hMSCs were seeded in tissue culture plates, and confluent cells were induced by the aforementioned culture medium in the presence of MDI (0.5 mmol L^−1^ IBMX, 1 µmol L^−1^ dexamethasone and 10 µg mL^−1^ insulin, all from Sigma) supplemented with or without recombinant GDF11 (rGDF11, PeproTech). The medium was changed every 3 days. 3T3‐L1 pre‐adipocytes were obtained from ATCC and seeded similar to hMSCs, except the culture medium. Instead of alpha‐MEM, 3T3‐L1 pre‐adipocytes were cultured in high‐glucose DMEM (Gibco). A pharmacological TGF‐beta type I receptor inhibitor, SB431542 (Selleck), was added to explore the underlying mechanisms.

### 2.2 Cytotoxicity measurement

Human mesenchymal stem cells were seeded in 96‐well tissue culture plates (2 × 10^4^ cells per well) and incubated for 24 hours in alpha‐MEM containing 10% FBS, 100 units mL^−1^ penicillin and 100 µg mL^−1^ streptomycin. And then, the cells were cultured in the medium containing different concentrations of rGDF11 for 48 hours. Cell viability was measured by the cell proliferation MTS kit (Promega). Briefly, the cells were washed with PBS for three times and refreshed with 200 µL medium per well. And 20 µL of 1.9 mg mL^−1^ 3‐(4,5‐dimethylthiazol‐2‐yl)‐5‐(3‐carboxymethoxyphenyl)‐2‐(4‐sulfophenyl)‐2H‐tetrazolium (MTS) solution was pipetted in each well and incubated for 4 hours. The absorbance was read at 490 nm in a microplate reader (Tecan). 3T3‐L1 pre‐adipocytes were seeded similar to hMSCs, except cultured in high‐glucose DMEM.

### 2.3 Oil Red O staining

Endocellular lipid accumulation was measured by Oil Red O (ORO) staining as previously described.^11^ In brief, after 2‐3 weeks of adipogenic induction, cells in 12‐well tissue culture plates were washed twice with pre‐cooled phosphate‐buffered saline (PBS; Gibco) and fixed in 4% paraformaldehyde (Biosharp) for 30 minutes. Cells were dyed with 0.5% ORO (Sigma) in 60% isopropanol for 30 minutes at room temperature after washed with 60% isopropanol. The cells stained with ORO were washed three times to remove the unbound excessive dye before being observed under the microscope. Then, the incorporated dye was extracted by 100% isopropanol (3 mL per well) and then diverted into a 96‐well tissue culture plate and quantified by absorbance values at 500 nm using spectrophotometer (Thermo Fisher Scientific).

### 2.4 Quantitative real‐time reverse transcription‐polymerase chain reaction(qRT‐PCR)

Total RNA of the cells was gathered employing TRIzol reagent (Invitrogen) on the basis of the protocol which was recommended by the manufacturer. The collected RNA was dissolved in DEPC water. 1.5% agarose gel electrophoresis was used to check the sample integrity, and NanoDrop 2000 (Thermo Fisher Scientific) was used to measure the concentration of total RNA. cDNA was prepared utilizing PrimeScript RT Reagent kit with gDNA Eraser (Takara). qRT‐PCR was performed using SYBR Premix Ex Taq II (Takara) on a Bio‐Rad CFX96 Real‐Time System. The sequences of primers are shown in Table 1. The relative mRNA expression of these genes was calculated employing the 2^−∆∆Ct^ method by standardizing with *36B4* housekeeping gene expression and compared to control.

**Table 1:** Primers for real‐time reverse transcription‐polymerase chain reaction

Genes	Primers	Sequences (5′‐3′)
*36B4*	Forward	AGCCCAGAACACTGGTCTC
	Reverse	ACTCAGGATTTCAATGGTGCC
*CD36*	Forward	GGCTGTGACCGGAACTGTG
	Reverse	AGGTCTCCAACTGGCATTAGAA
*PLIN1*	Forward	TGTGCAATGCCTATGAGAAGG
	Reverse	AGGGCGGGGATCTTTTCCT
*ADIPOQ*	Forward	CCCTCTCTTACAAGCCCATCA
	Reverse	GAGCCAGTCTGGTAGTACATCA
*PPARG*	Forward	ACCAAAGTGCAATCAAAGTGGA
	Reverse	ATGAGGGAGTTGGAAGGCTCT
*CEBPA*	Forward	TTCACATTGCACAAGGCACT
	Reverse	GAGGGACCGGAGTTATGACA
*LPL*	Forward	TCATTCCCGGAGTAGCAGAGT
	Reverse	GGCCACAAGTTTTGGCACC
*36B4 (mouse)*	Forward	TGAGATTCGGGATATGCTGTTGG
	Reverse	CGGGTCCTAGACCAGTGTTCT
*Cd36*	Forward	GAGCAACTGGTGGATGGTTT
	Reverse	GCAGAATCAAGGGAGAGCAC
*Plin1*	Forward	CCTGTGGTGAGCGGGACC
	Reverse	GTGGACAGCCGACGGACC
*Adipoq*	Forward	CGTCACTGTTCCCAATGT
	Reverse	ACCGTGATGTGGTAAGAG
*Pparg*	Forward	CATCAGGCTTCCACTATG
	Reverse	CACAGCAAGGCACTTCTG
*Cebpa*	Forward	ACTCCTCCTTTTCCTACCG
	Reverse	AGGAAGCAGGAATCCTCC
*Lpl*	Forward	GGGAGTTTGGCTCCAGAGTTT
	Reverse	TGTGTCTTCAGGGGTCCTTAG

### 2.5 Western blot

Cells were disposed with different concentrations of rGDF11 with or without SB431542 at 37°C for 30 minutes prior to sampling. As described previously,^12^ cells were lysed in RIPA buffer (Pierce) on ice. The samples were heated at 95°C for 5 minutes in sample buffer containing 1% 2‐mercaptoethanol and 2% SDS, separated on 10% SDS‐polyacrylamide gels and transferred to PVDF membranes (Millipore) by a Bio‐Rad wet transfer apparatus. The membranes were blocked with 5% BSA for 1 hour and then incubated overnight with primary antibodies: rabbit anti‐phospho‐Smad2 (1:1000; Thermo Fisher), rabbit anti‐phospho‐Smad3 (1:1000; Cell Signaling), rabbit anti‐Smad2/3 (1:1000; Cell Signaling) and α‐tubulin rabbit polyclonal antibody (1:2000; Beyotime). The immunocomplexes were incubated with a goat anti‐rabbit IgG secondary antibody HRP conjugated (1:5000; Cell Signaling). The antibody‐antigen complexes were visualized with Immobilon reagents (Millipore).

### 2.6 Immunofluorescence staining

Cells were adhered on clean coverslips in 24‐well tissue culture plates. After reaching 60%‐70% confluence, cells were disposed with different concentrations of rGDF11 with or without SB431542 at 37°C for 30 minutes prior to sampling. Cells were washed with pre‐cooled PBS for three times and fixed in 4% paraformaldehyde for 10 minutes at room temperature. After that, the cells were washed with PBS again and transferred to glass slides. Then, the cells were blocked with 4% BSA at room temperature for 30 minutes. Cells were incubated with primary antibodies: rabbit anti‐phospho‐Smad2 (1:800) and rabbit anti‐phospho‐Smad3 (1:250) at 4°C overnight, and then stained with Alexa Fluor 555 (1:200; Invitrogen), respectively. DAPI (Vector) was used as counterstain.

### 2.7 Chromatin immunoprecipitation assay

Chromatin immunoprecipitation (ChIP) assays were performed employing a SimpleChIP Assay kit (Cell Signaling Technology) according to the manufacturer's protocol, as described previously.^9^ Immunoprecipitation was performed using antibodies directed against histone deacetylase 1 (HDAC1) (Abcam) and rabbit IgG (Millipore) as a negative control. Precipitated DNA samples were quantified with real‐time PCR using PPARγ primer, forward, 5′‐GAGCAAGGTCTTCATCATTACG‐3′; reverse, 5′‐CCCCTGGAGCTGGAGTTAC‐3′. Data were presented as the percentage of input DNA.

### 2.8 Statistics

All values were presented as the mean ± SEM. Two‐tailed Student's *t* test was used for comparison between two groups, and a one‐way analysis of variance (ANOVA) followed by a Tukey's post hoc test was performed for multiple comparisons. A *P*‐value of <0.05 was considered to be statistically significant.

## 3 RESULTS

### 3.1 GDF11 impairs adipogenic differentiation of human MSCs

**Figure 1:**
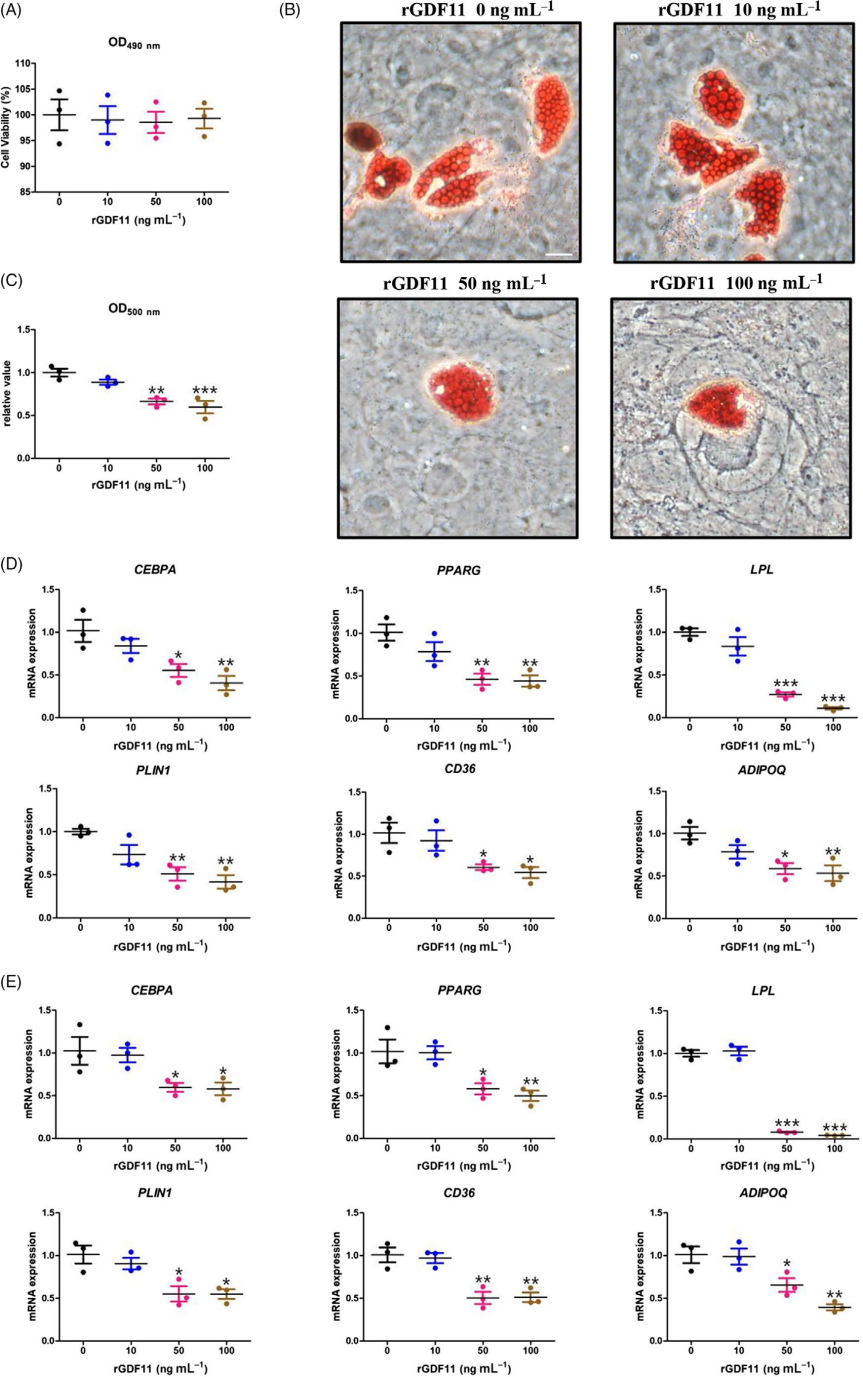
GDF11 impairs adipogenic differentiation of human MSCs. A, MTS assay of hMSCs treated with different concentrations of rGDF11. B, Oil Red O staining 21 d after adipogenic differentiation. Scale bar, 25 μm. C, Quantification of lipid accumulation in hMSCs supplemented with rGDF11. Triglyceride content was measured at 500 nm after extracting Oil Red O. D, qRT‐PCR analysis revealed reduced mRNA expressions of adipocyte‐specific molecular markers *PPARG*,* CEBPA*,* LPL*,* PLIN1*,* CD36* and *ADIPOQ* 7 d after differentiation in high concentrations (50 and 100 ng mL^−1^) of rGDF11‐treated hMSCs. E, qRT‐PCR analysis revealed reduced mRNA expressions of adipocyte‐specific molecular markers *PPARG*,* CEBPA*,* LPL*,* PLIN1*,* CD36* and *ADIPOQ* 14 d after differentiation in high concentrations (50 and 100 ng mL^−1^) of rGDF11‐treated hMSCs. n = 3. *P < 0.05, **P < 0.01, ***P < 0.001. Results were shown as mean ± SEM, ANOVA

First, to investigate the cytotoxicity of rGDF11, hMSCs were treated with various concentrations (10‐100 ng mL^−1^) of rGDF11 and the cell viability was monitored using an MTS assay. We observed that treatment with 10‐100 ng mL^−1^ of rGDF11 did not have any significant cytotoxic effects on hMSCs (Figure 1A).

To explicit the effect of GDF11 on adipogenic differentiation, hMSCs were induced in the presence of MDI with graded concentrations of rGDF11. As visualized by ORO staining, the supplement of rGDF11 diminished lipid accumulation after 21 days of induction (Figure 1B). Quantitative analyses further confirmed that although low dosage of rGDF11 (10 ng mL^−1^) was insufficient to significantly reduce accumulated triglyceride, higher doses of rGDF11 (50 ng mL^−1^) were already enough to do so, while the highest concentration of rGDF11 (100 ng mL^−1^) achieved the strongest inhibitory effect with almost 40% reduction (Figure 1C).

To confirm this negative effect of GDF11 on hMSC adipogenic differentiation on transcription levels, quantitative reverse transcription‐polymerase chain reaction (qRT‐PCR) was carried out at both an early stage (treated 7 days) and a terminal phase (treated 14 days) of adipogenic differentiation (Figure 1D,E). Similar to ORO staining, only higher doses of rGDF11 (50 and 100 ng mL^−1^) were able to significantly downregulate mRNA levels of *CEBPA* and *PPARG*, two master adipogenic transcription markers, along with other adipogenic‐related genes *LPL*,* PLIN1*,* CD36* and *ADIPOQ* at both early and terminal phases. Taken together, both morphologic and transcription‐level results implicate that GDF11 restrains adipogenic differentiation of hMSCs.

### 3.2 GDF11 inhibits adipogenic differentiation of 3T3‐L1 pre‐adipocytes

**Figure 2:**
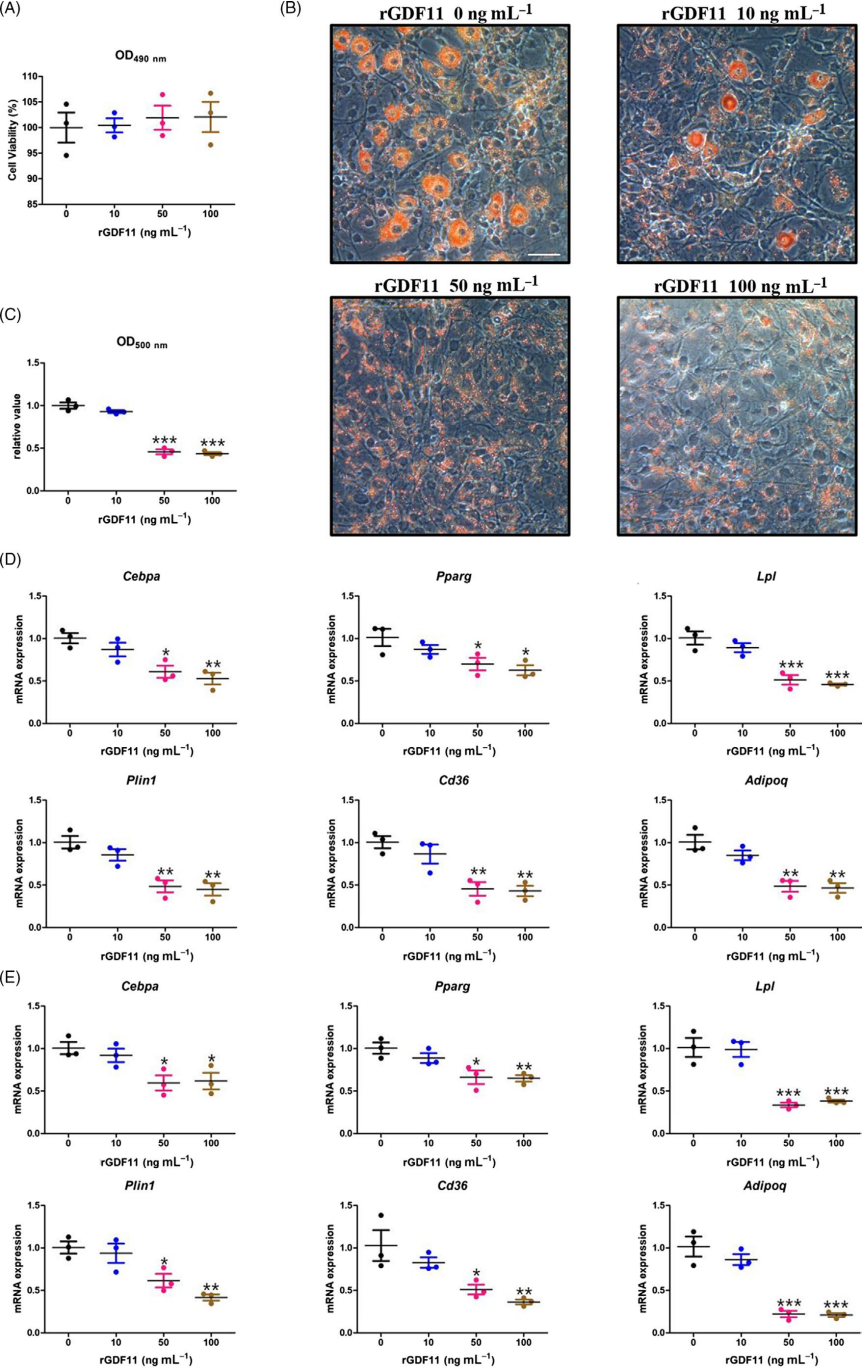
GDF11 inhibits adipogenic differentiation of 3T3‐L1 pre‐adipocytes. A, MTS assay of 3T3‐L1 pre‐adipocytes treated with different concentrations of rGDF11. B, Oil Red O staining of 3T3‐L1 pre‐adipocytes 7 d after adipogenic differentiation. Scale bar, 25 μm. C, Quantification of lipid accumulation in of 3T3‐L1 pre‐adipocytes supplemented with rGDF11. Triglyceride content, measured at 500 nm after extracting Oil Red O, was diminished in 50 and 100 ng mL^−1^ rGDF11‐treated groups. D, qRT‐PCR results. The relative mRNA expressions of adipocyte‐specific molecular markers *Pparg, Cebpa*,* Lpl, Plin1*,* Cd36* and *Adipoq* were analysed on Day 3 after differentiation. E, qRT‐PCR results. The relative mRNA expressions of adipocyte‐specific molecular markers *Pparg, Cebpa*,* Lpl, Plin1*,* Cd36* and *Adipoq* were analysed on Day 5 after the differentiation. n = 3. *P < 0.05, **P < 0.01, ***P < 0.001. Results were shown as mean ± SEM, ANOVA

To validate this negative effect on adipogenic‐committed cells, we conducted the same experiments in 3T3‐L1 pre‐adipocyte cell line. In line with hMSCs, cell viability was measured first, and the presence of 10‐100 ng mL^−1^ of rGDF11 did not exert any significant cytotoxic effects on 3T3‐L1 pre‐adipocytes either (Figure 2A).

In accordance with hMSCs, diminished lipid accumulation was observed in 50 and 100 ng mL^−1^ rGDF11‐treated groups 7 days after induction, as visualized by ORO dye (Figure 2B) and quantified by spectrophotometry (Figure 2C). While the lower concentration of rGDF11 (10 ng mL^−1^) only slightly inhibited adipogenic differentiation potency, being insignificantly different from control group (*P* = 0.1565). In line with ORO staining data, qRT‐PCR analyses revealed a significant reduction in mRNA levels of *Pparg*,* Cebpa*,* Lpl*,* Cd36*,* Plin1* and *Adipoq* in the higher concentrations of rGDF11 (50 and 100 ng mL^−1^), but not in the lower one (10 ng mL^−1^) compared with controls (Figure 2D,E).

### 3.3 GDF11 stimulates the phosphorylation of Smad2/3 during 3T3‐L1 adipogenesis

**Figure 3:**
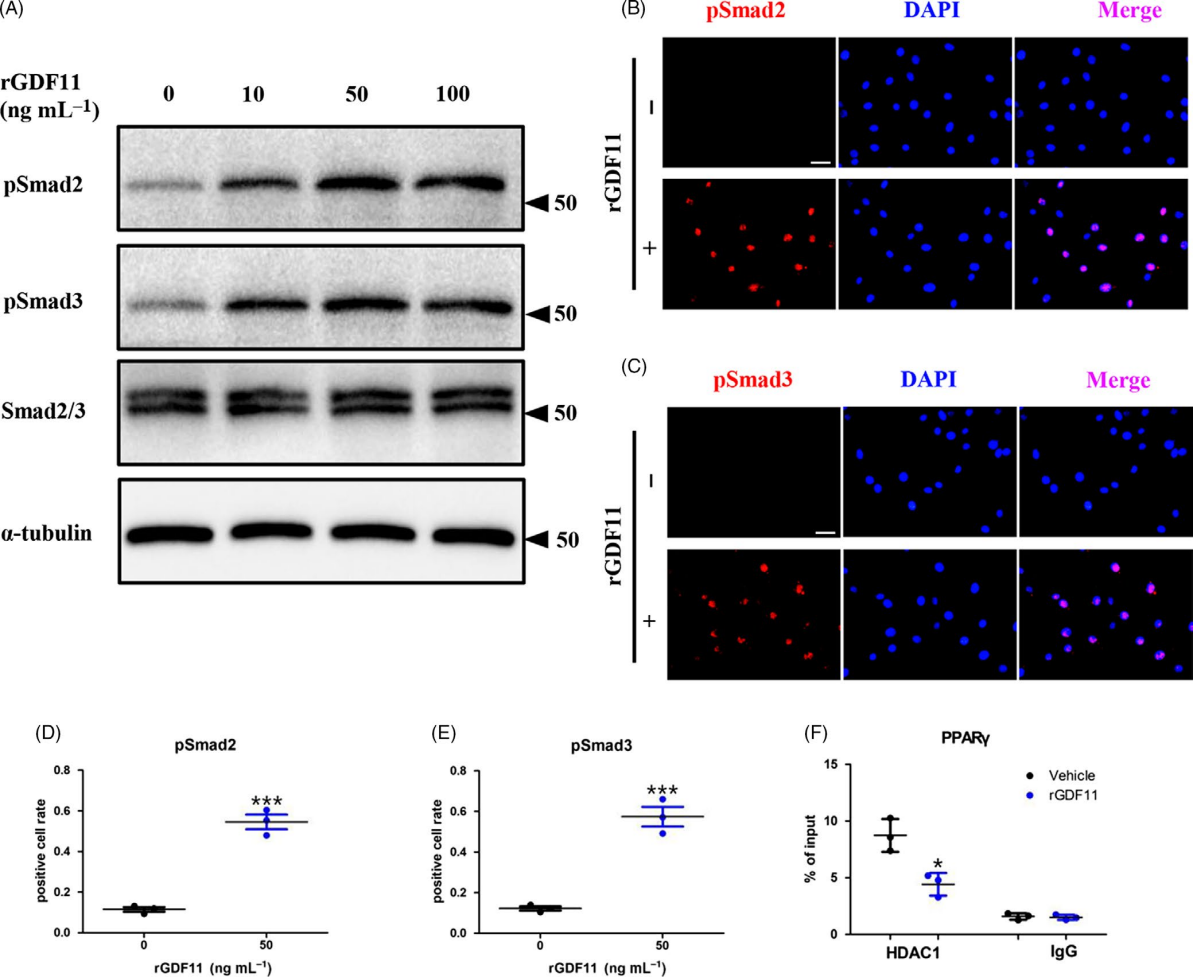
GDF11 activates Smad2/3‐dependent TGF‐beta signalling pathway. A, Western blot analysis revealed that GDF11 stimulated the phosphorylation of Smad2 and Smad3. B, Immunofluorescence staining of 3T3‐L1 pre‐adipocytes indicated that GDF11 activated the phosphorylation of Smad2 in 30 min. Scale bar, 1 mm. C, Cell immunofluorescence analysis demonstrated that GDF11 increased pSmad3‐positive cells in 3T3‐L1 pre‐adipocytes cultured under adipogenic conditions. Scale bar, 1 mm. D, Quantification of the pSmad2‐positive cell in (B). E, Quantification of the pSmad3‐positive cell in (C). F, ChIP assay revealed that rGDF11 reduced the abundance of HDAC1 at the CEBP binding site of the PPARγ promoter. n = 3. *P < 0.05. ***P < 0.001. Results were shown as mean ± SEM, *t* test

To elucidate the underlying mechanisms that drive the inhibitory effects of GDF11 on adipogenic differentiation, we then checked whether TGF‐beta/Smad signalling pathway was involved. Western blot analysis confirmed on protein level that GDF11 did stimulate the phosphorylation of Smad2 and Smad3 in 3T3‐L1 pre‐adipocytes, without changing the amount of Smad2/3 (Figure 3A). Immunofluorescence staining demonstrated that the presence of rGDF11 (50 ng mL^−1^) significantly increased pSmad2 (Figure 3B)‐ and pSmad3 (Figure 3C,E)‐positive cells in 3T3‐L1 pre‐adipocytes cultured under adipogenic conditions. Previous reports showed that the activation of Smad3 represses C/EBP transactivation function by inducing HDAC1 at the PPARγ promoter.^13^, ^14^, ^15^ In addition, our ChIP assay demonstrated that rGDF11 significantly decreased the abundance of HDAC1 at the C/EBPs binding site of the PPARγ promoter (Figure 3F).

### 3.4 ALK4,5 inhibitors attenuate the effect of GDF11 on adipogenic differentiation

**Figure 4:**
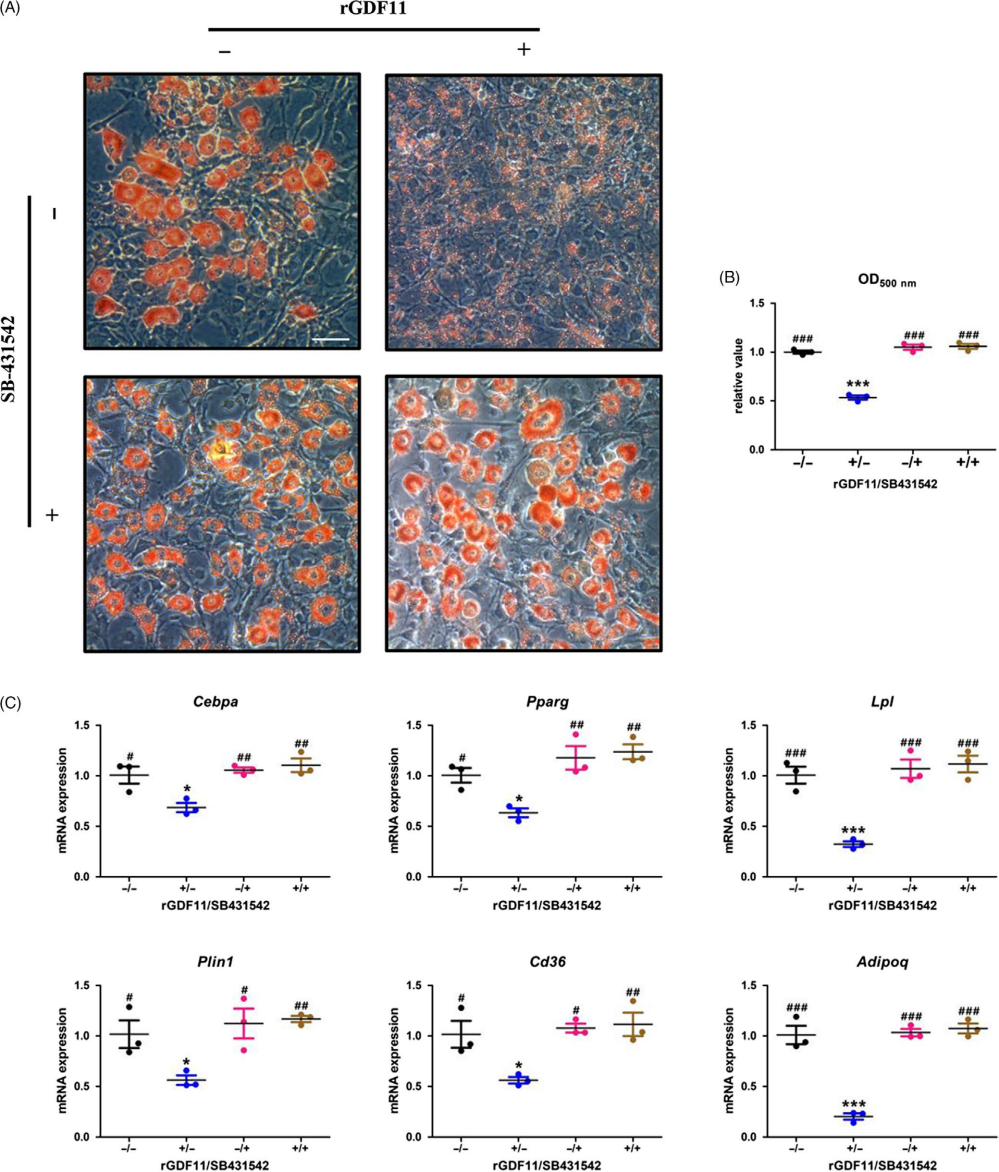
ALK4,5 inhibitors attenuate the effect of GDF11 on adipogenic differentiation. A, 3T3‐L1 cells were fixed and stained with Oil Red O 7 d after differentiation. The presence of SB431542 recovered the number of red‐stained cells which was inhibited by rGDF11. Scale bar, 25 μm. B, Triglyceride content was measured at 500 nm after extracting Oil Red O. Lipid accumulation diminished by rGDF11 was fully restored by SB431542. C, qRT‐PCR results. The relative mRNA expressions of adipocyte‐specific molecular markers *Pparg, Cebpa*,* Lpl, Plin1*,* Cd36* and *Adipoq* were analysed on 3 d after differentiation. n = 3. *P < 0.05 vs GDF11(−)/SB431542(−), **P < 0.01 vs GDF11(−)/SB431542(−), ***P < 0.001 vs GDF11(−)/SB431542(−). ^#^ *P* < 0.05 vs GDF11(+)/SB431542(−), ^##^ *P* < 0.01 vs GDF11(+)/SB431542(−), ^###^ *P* < 0.001 vs GDF11(+)/SB431542(−). Results were shown as mean ± SEM, ANOVA

To further validate whether the inhibited adipogenesis is mediated by GDF11, we combined SB‐431542, the potent and specific inhibitor of TGF‐beta type I activin receptor‐like kinase (ALK) receptors, along with GDF11 to treat 3T3‐L1 pre‐adipocytes under adipogenic medium.^16^ The presence of SB431542 fully recovered the adipogenic potential inhibited by rGDF11 in 3T3‐L1 pre‐adipocytes as clearly demonstrated by ORO staining (Figure 4A) and quantitative analyses (Figure 4B). Furthermore, SB431542 successfully restored adipogenic‐related gene expression (Figure 4C,D).

### 3.5 ALK4,5 inhibitors eliminate GDF11‐induced phosphorylation of Smad2/3

**Figure 5:**
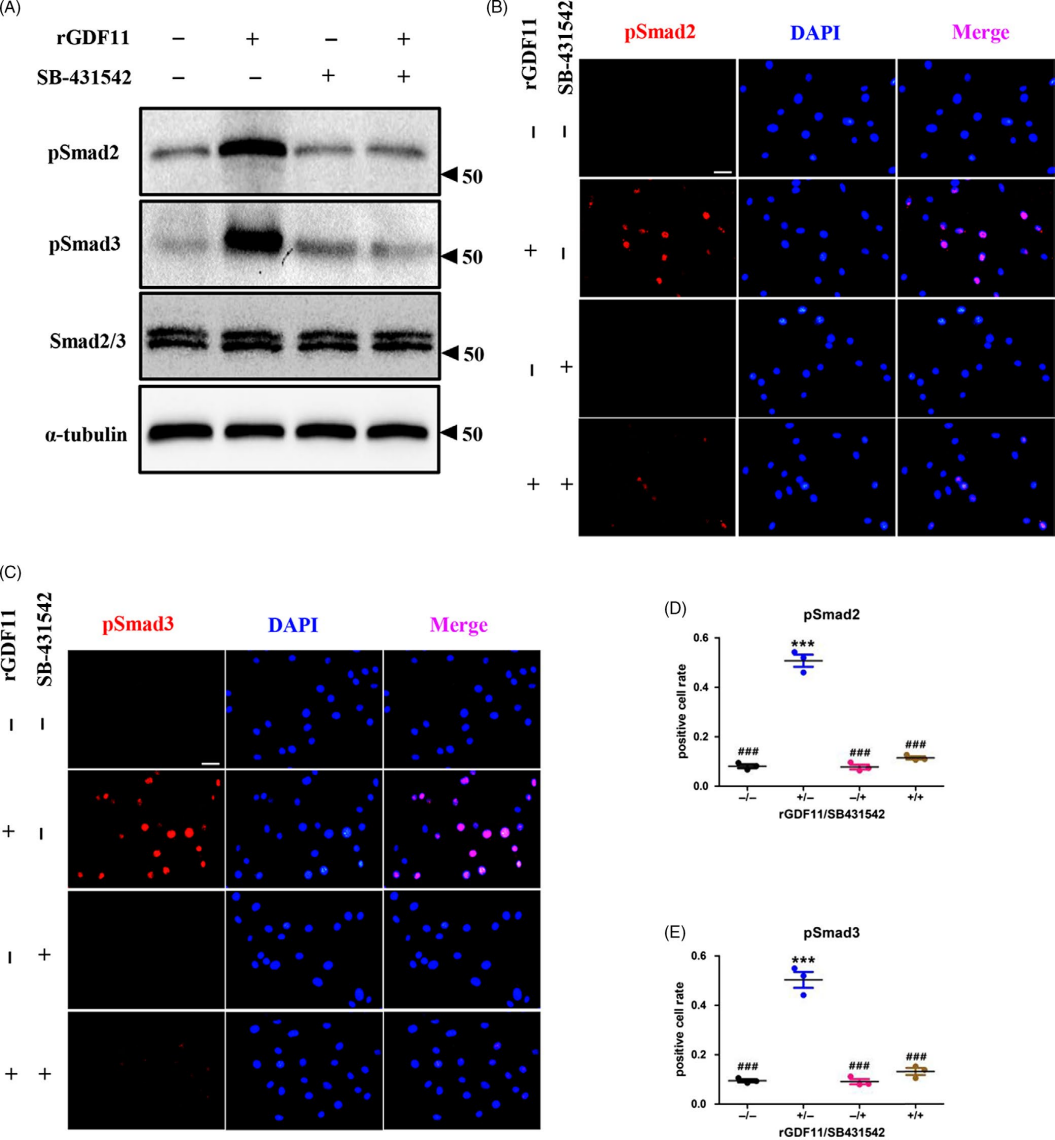
ALK4,5 inhibitors eliminate GDF11‐induced phosphorylation of Smad2/3. A, Western blot analysis indicated that the phosphorylation of Smad2/3 by rGDF11 was totally blocked by SB431542. B, Cell immunofluorescence analysis showed that rGDF11 failed to increase the number of pSmad2‐positive cells under the treatment of SB431542. Scale bar, 1 mm. C, Cell immunofluorescence analysis demonstrated that SB431542 diminished the rGDF11‐activated Smad3 phosphorylation. Scale bar, 1 mm. D, The positive cell rate in (B). E, The positive cell rate in (C). n = 3. ***P < 0.001 vs GDF11(−)/SB431542(−). ^###^ *P* < 0.001 vs GDF11(+)/SB431542(−). Results were shown as mean ± SEM, ANOVA

Finally, to confirm whether the activation of Smad2/3 is enforced by GDF11, we supplemented the culture medium with SB‐431542. Western blot analyses showed that pSmad2 and pSmad3 expression was upregulated by rGDF11, and these activation effects were totally blocked by SB431542 (Figure 5A). Immunofluorescence staining (Figure 5B‐E) demonstrated that GDF11 failed to increase the number of pSmad2‐ or pSmad3‐positive cells when treated with SB431542.

## 4 DISCUSSION

Bone marrow‐derived human mesenchymal stem cells have been established as multipotent progenitor cells, which possess the potential to differentiate into osteogenic, chondrogenic and adipogenic progenitors, and eventually multilineages of mesenchymal tissues, such as bone, cartilage, fat, muscle and tendon.^17^ It was well characterized that hMSC‐derived adipocytes have common adipogenic lineage‐specific gene expression pattern parallel to primary adipocytes,^18^ thus qualifying hMSCs a valid in vitro model to investigate the effects of GDF11 on adipogenic lineage commitment. Preceding projects of our laboratory have been focused on the osteogenic and chondrogenic differentiation of MSCs.^9^, ^10^ To understand the role of GDF11 in MSC lineage commitment more comprehensively, we initiate the current project on adipogenic potential.

GDF11 is widely distributed in both embryonic and adult tissues in human with varying mRNA and protein levels among different organs. To study the role of exogenous GDF11 in vitro, 50 ng mL^−1^ of rGDF11 was employed to investigate its function to prevent cardiac hypertrophy in cardiomyocytes,^19^ to induce kidney fibrosis in renal cell lines^20^ and to improve angiogenic in endothelial progenitor cells.^21^ In our previous projects, graded concentrations of 0, 20, 50 and 100 ng mL^−1^ were used to study its function in osteoclastogenesis in bone marrow macrophages, as well as osteoblast differentiation in mouse MSCs and calvarial osteoblasts.^9^ And 0, 10 and 50 ng mL^−1^ were applied to examine its role in mouse MSCs during chondrogenesis.^10^ In the present experiment, 0, 10, 50 and 100 ng mL^−1^ were chosen to examine the possible impact of exogenous GDF11 in hMSCs and 3T3‐L1 pre‐adipocytes.

We found that only high concentrations (50 and 100 ng mL^−1^) of rGDF11 were able to impair adipogenic differentiation significantly, while 10 ng mL^−1^ was insufficient to do so in hMSCs. The mRNA levels of *PPARG*, which is both necessary and sufficient for adipogenesis,^22^ were dose‐dependently downregulated by GDF11. *PPARG* and *CEBPA* are recognized as two principle adipogenic transcription factors that regulate the promoters of downstream adipogenic‐related genes and activate full differentiation process required for adipocyte maturation.^22^ It was worth noticing that *LPL*, which is involved in adipocyte fat uptake and storage, was the one with most dramatic changes upon rGDF11 administration, indicating GDF11 might be exerting negative effects through regulating fat metabolism. Expression of other adipogenic‐related genes, such as *CD36*,* PLIN1* and *ADIPOQ*, was also reduced. So collectively, GDF11 did exert negative effects on adipogenesis in hMSCs.

TGF‐beta superfamily members have been shown to play critical roles in regulating adipogenesis; for instance, TGF‐beta itself inhibits adipogenic differentiation only when it is added before commitment point.^23^ This interesting finding intrigued us to test whether GDF11 could exert similar effects on committed precursors. 3T3‐L1 pre‐adipocyte, which is already committed to adipogenic lineage, turned out to be the ideal cell line to decipher the underlying mechanism.^24^ Both ORO staining and qRT‐PCR results evidenced that GDF11 suppresses the terminal differentiation of 3T3‐L1 pre‐adipocytes into mature adipocytes.

Hereby, we have shown that GDF11 suppresses adipogenesis in both hMSCs and pre‐adipocytes. Yet, the mechanism by which these inhibitory effects were orchestrated remains vague. Accumulating evidence has suggested that GDF11 and GDF8 (also known as myostatin), which share 90% sequence identity in their mature region, primarily use ALK4 and ALK5 to elicit downstream intracellular signalling cascade by SMAD proteins.^25^ Smad2/3 activation had been proven to be critical in mediating inhibitory effects of myostatin in C3H10T1/2, 3T3‐L1^26^ and hMSC adipogenesis.^27^ In addition, our laboratory showed earlier that GDF11 rapidly induces Smad2/3 phosphorylation in both BMMs and primary osteoblasts.^9^ However, it is not clear whether this canonical Smad‐dependent signal pathway is activated by GDF11 during adipogenesis. Western blot analysis showed that GDF11 increased the protein levels of pSmad2 and pSmad3, without changing Smad2/3. Immunofluorescence staining also showed increased number of pSmad2‐ and pSmad3‐positive cells by GDF11. Hence, our data verified that GDF11 did increase the phosphorylation of Smad2/3 during adipogenic differentiation in 3T3‐L1 pre‐adipocyte, indicating that GDF11 signals through a Smad2/3‐dependent TGF‐beta pathway.

Last, to determine whether the phosphorylation of Smad2/3 is indeed elicited by GDF11, we supplement the adipogenic culture medium with SB‐431542, a specific inhibitor of ALK4, ALK5 and ALK7, in addition to rGDF11. ORO and qRT‐PCR results showed that the administration of SB‐431542 totally restored lipid formation and adipogenic‐related gene expression pattern repressed by rGDF11. The successful restoration by SB‐431542 implies that the inhibition of adipogenesis by GDF11 is mediated through the receptors blocked by SB‐431542, that is, ALK4, ALK5 and ALK7. Besides GDF11, several other GDFs, including GDF1, GDF3, GDF8 and GDF9, also bind with ALK4, ALK5 or ALK7.^26^, ^28^, ^29^ During embryogenesis, GDF11 has been reported to mainly interact with ALK4 and ALK5 to activate Smad3‐dependent reporter gene to regulate anterior‐posterior patterning.^30^ Receptor utilization has been found to be cell type–specific, in C2C12 myoblasts, GDF8 predominately utilizes ALK4, while it prefers ALK5 in C3H1‐T1/2 and 3T3‐L1.^31^ Further investigation is needed to delineate the receptor utilization of GDF11 in 3T3‐L1 during adipogenesis.

Mechanically, we found that SB‐431542 was able to completely restore the activation of Smad2/3 in 3T3‐L1 cell line. It is worth noting that the adipogenic potential of 3T3‐L1 pre‐adipocytes treated with only SB‐431542 in the medium was comparable to that of control, implying that the inhibitor itself exerted neither positive nor negative effect on adipogenesis.

In summary, we demonstrated that GDF11 inhibits adipogenesis in vitro in both hMSCs and 3T3‐L1 adipocytes. GDF11 significantly reduced lipid accumulation and adipogenic‐related gene expression in a concentration‐dependent manner at both the early and terminal stages during adipogenic differentiation. Mechanically, we showed that these inhibitory effects were mediated through the increased phosphorylation of Smad2/3 and could be completed restored by SB‐431542, a TGF‐beta type I receptor inhibitor. Our data demonstrate that GDF11 is a regulator of adipogenic differentiation.

## CONFLICT OF INTEREST

The authors declare that there is no conflict of interest regarding the publication of this paper.
